# Serum kisspeptin levels in pregnant and non-pregnant diestrus bitches: a pilot study

**DOI:** 10.3389/fvets.2026.1826654

**Published:** 2026-04-02

**Authors:** Temy Coppola, Olimpia Barbato, Laura Menchetti, Gabriele Brecchia, Andrea Verini Supplizi, Giovanni Ricci, Sabrina Caloiero, Viola Zappone, Marco Quartuccio, Santo Cristarella, Angela Polisca, Alessandro Troisi

**Affiliations:** 1 Department of Veterinary Medicine, University of Perugia, Perugia, Italy; 2 Department of Biosciences, University of Camerino, Camerino, Italy; 3 Kennel Training Course of Financial Guard, Castiglione del Lago, Italy; 4 Department of Veterinary Medicine, University of Messina, Messina, Italy

**Keywords:** bitch, early biomarker, kisspeptin, placenta, pregnancy

## Abstract

Kisspeptins are neuropeptides that play a key role in regulating reproductive function. They are also involved in maternal-fetal communication and the development of the placenta in several mammalian species. In humans and cattle, circulating kisspeptin concentrations increase during pregnancy, and the placenta has been proposed as their main source of production. To the best of the authors’ knowledge, no data are currently available regarding serum kisspeptin concentrations during canine pregnancy. The aim of this study was to evaluate, for the first time, serum kisspeptin concentrations in pregnant bitches compared with non-pregnant diestrus bitches. Eight clinically healthy German Shepherd bitches were enrolled and divided into two groups: pregnant (*n* = 4) and non-pregnant diestrus (*n* = 4). Blood samples were collected from ovulation and every 15 days until the end of pregnancy or diestrus. Pregnancy was diagnosed using ultrasonography at 19 days post-ovulation and was monitored simultaneously with blood sampling. Median serum kisspeptin concentrations throughout the observation period tended to be higher in pregnant bitches, with a statistically significant difference between groups observed on Day 15 post-ovulation (*p* = 0.029). This early increase in serum kisspeptin concentrations suggests that kisspeptin circulating in the bloodstream deserves further investigation as a potential biomarker for the early detection of pregnancy in bitches. However, these findings should be interpreted with caution given the exploratory nature of the study and its small sample size.

## 1 Introduction

Kisspeptin (KP) is a neuropeptide that was originally isolated from a human melanoma cell line. There, it was identified as a metastasis suppressor and named “metastin” (1). Kisspeptin is encoded by the KISS1 gene, and it exerts its biological activity by binding to the G protein-coupled receptor KISS1R, which was previously known as GPR54 (2). Located on chromosome 1q32, the KISS1 gene encodes a 145-amino-acid precursor peptide that undergoes post-translational processing to generate several biologically active fragments, including KP-54, KP-14, KP-13 and KP-10 (2–4).

The kisspeptinergic system is widely recognized as a key regulator of reproductive function, primarily due to its involvement in the hypothalamic-pituitary-gonadal (HPG) axis (5–7). Kisspeptins stimulate gonadotropin-releasing hormone (GnRH) neurons by activating KISS1R, thereby promoting GnRH synthesis and secretion (8). GnRH then stimulates pituitary gonadotrophs to release follicle-stimulating hormone (FSH) and luteinising hormone (LH), which regulate gonadal function (9–11). The importance of this system is emphasised by evidence showing that inactivating mutations in KISS1 or KISS1R lead to hypogonadotropic hypogonadism (5–7), whereas activating mutations in KISS1R have been associated with precocious puberty (12).

In addition to its role in central neuroendocrine regulation, the kisspeptinergic system is also expressed in the peripheral reproductive tissues of both males and females. In female mammals, both kisspeptin and its receptor have been detected in ovarian tissues of various species, including rats, mice, humans, dogs, pigs, cattle and cats (13–30). This suggests that kisspeptin plays an autocrine and paracrine role in reproductive processes. Furthermore, kisspeptins are involved in pregnancy-related processes. In humans, KISS1 is expressed in placental syncytiotrophoblasts, whereas KISS1R is found in both cytotrophoblasts and syncytiotrophoblasts (31). The kisspeptinergic system has also been documented in the placenta of other species, including dogs and cattle (32–34).

Placenta kisspeptins are thought to contribute to several processes involved in pregnancy establishment and maintenance. They are involved in processes such as embryo implantation, trophoblast invasion, placental angiogenesis and maternal immune adaptation (31, 35–38). Circulating kisspeptin concentrations increase markedly due to placental production during gestation. In women, serum kisspeptin levels progressively rise throughout pregnancy, reaching concentrations over 900 times higher than in non-pregnant individuals in the first trimester, and over 7,000 times higher in the third trimester. This is followed by a rapid decline after parturition (39). Similar increases have been reported in cattle, although temporal variations among gestational stages have also been observed, suggesting the existence of species-specific regulatory mechanisms (27, 40).

Despite growing knowledge of the role of kisspeptin in the reproductive physiology of various species, there is a lack of information on circulating kisspeptin concentrations during pregnancy in female dogs. The present study aimed to determine serum kisspeptin concentrations during canine pregnancy and compare them with those observed in non-pregnant bitches in diestrus. This comparison was conducted to investigate the potential role of this neuropeptide during canine gestation. Therefore, this study should be considered an initial exploratory investigation, designed to generate hypotheses regarding the potential involvement of kisspeptin in the early stages of canine pregnancy.

## 2 Materials and methods

### 2.1 Ethical approval

All treatments, housing and animal care followed EU Directive 2010/63/EU on the protection of animals used for scientific purposes. The Ethics Committee of the Department of Veterinary Medicine and Animal Productions at the University of Perugia, Italy (prot. no. 314949), approved the protocol and procedures. Informed consent was obtained from each dog owner before its inclusion in the study.

### 2.2 Animals and study design

Eight clinically healthy female German Shepherd dogs were enrolled in the study and divided into two groups: a pregnant group (*n* = 4) and a non-pregnant group (*n* = 4). The bitches ranged in age from 2 to 4 years and weighed 29 ± 1 kg. All animals were born and raised at the Canine Training and Breeding Center of the Guardia di Finanza (Castiglione del Lago, Perugia, Italy). The bitches assigned to the pregnant group were multiparous and had a regular reproductive history, having previously completed at least two pregnancies that resulted in the normal delivery of viable, healthy puppies. In contrast, the bitches included in the non-pregnant group were in physiological cyclical oestrus and were not mated during the study period, as they were being monitored within a controlled breeding selection programme for their prospective use as brood bitches. These animals had previously exhibited two to three normal oestrous cycles but had not yet experienced pregnancy.

All animals underwent thorough clinical and reproductive examinations, including anamnesis, medical history, physical examination, and routine hematological and biochemical analyses. They were fed twice daily (08:00 and 20:00) a commercial, complete, and balanced diet for reproduction (REPRODUCTION FEMALE dry food, Sanypet S.p.a., Bagnoli Di Sopra (PD), Italy). The diet was provided according to physiological state, with an estimated average energy demand of 132 kcal/kg BW^0.75^ for pregnant bitches until day 40 and for non-pregnant bitches throughout the study.

### 2.3 Sample collection and reproductive monitoring

The day of ovulation was designated as “Day 0,” which was confirmed when serum progesterone concentrations were between 4 and 10 ng/mL (41–43). In the bitches assigned to the pregnant group, natural mating was allowed approximately 48 h after ovulation. Blood samples were collected from the cephalic vein on Day 0 and subsequently every 15 days until the end of pregnancy or the luteal phase. The sampling days 15, 30, 45, and 60 post-ovulation were designated as Day 1, Day 2, Day 3, and Day 4, respectively. The end of diestrus was defined as the day when serum progesterone concentrations fell to ≤2 ng/mL. The ovulation day was also confirmed by transabdominal ultrasonography (My Lab 30 Gold Esaote, Genova, Italy), as well as pregnancy diagnosis on Day 19 post-ovulation. Embryo-fetal development was monitored on the same days as blood sampling (Days 1, 2, 3, and 4). For all animals, an aliquot of each blood sample was immediately analyzed for hematological and biochemical parameters. The remaining serum was separated and stored at −80 °C until hormonal assay.

### 2.4 Hormonal assays

Serum concentrations of kisspeptin and progesterone were determined using commercial enzyme immunoassay (ELISA) kits according to the manufacturers’ instructions. All samples were assayed in duplicate. Kisspeptin concentrations were measured using a Canine Kisspeptin ELISA Kit (MyBioSource, Catalog MBS7262277, San Diego, CA, United States). According to the manufacturer’s specifications and our in-house validation, the assay sensitivity was 1.0 pg/mL. The mean recovery rate ranged from 94 to 103%. The intra-assay and inter-assay coefficients of variation (CV) were 4.4–5.6% and 6.6–7.9%, respectively. Progesterone serum concentrations were measured using a commercial Progesterone ELFA Kits (MiniVidas bio-Merieux) validated for canine (44).

### 2.5 Statistical analysis

Due to the number of animals examined in this study, the data were presented as medians, range (minimum–maximum), and interquartile range. The two groups were compared using a highly robust non-parametric approach, namely the Independent-Samples Median Test (Fisher’s exact test). In addition, effect size (*r*) was calculated from the Mann–Whitney *U* test as *Z*/√*N*. The Hodges–Lehmann estimator and corresponding 95% confidence intervals were used to quantify differences between groups. Changes over time within each group were analyzed using the Friedman test, with effect size estimated by Kendall’s *W*. A *p*-value ≤0.05 was considered statistically significant. All analyses were performed using IBM SPSS Statistics (version 27, IBM Corp., Armonk, NY, United States) and graphical representations were generated using GraphPad Prism (version 8, GraphPad Software, San Diego, CA, United States).

## 3 Results

All pregnant bitches delivered healthy puppies (mean litter size 9.75 ± 4.57). Non-pregnant diestrus females exhibited physiological progesterone levels (≥5 ng/mL) (45).

**Figure 1:**
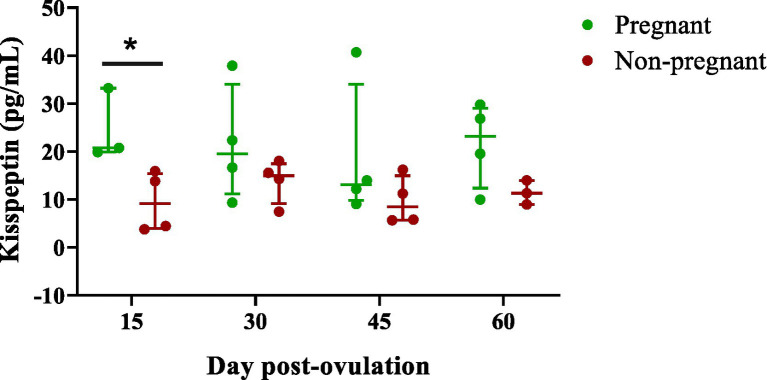
Interval scatter plot showing median kisspeptin concentrations and interquartile range in pregnant and non-pregnant bitches across the four post-ovulation days. *p < 0.05.

The descriptive statistics and the inferential test results are reported in Table 1 and in the interval scatter plot (Figure 1). A statistically significant difference between groups was detected only on Day 1, with pregnant bitches showing markedly higher values than non-pregnant ones (*p* = 0.029), with a Hodges–Lehmann estimate of −16.2 (95% CI: −29.4 to −4.0) and a large effect size (*r* = 0.80). Although there was no statistical significance on the remaining days, the different ranges and maximum values between the two groups were still evident. On Day 2, the maximum value in non-pregnant bitches was 18.1 pg/mL, whereas in pregnant bitches it reached 37.9 pg/mL (*p* = 0.486; Hodges–Lehmann estimate = −5.5, 95% CI: −23.6 to 6.2; *r* = 0.41). On Day 3, the maximum in the non-pregnant group was 16.2 pg/mL, whereas in the pregnant group it was more than double this at 40.7 pg/mL (*p* = 0.486; Hodges–Lehmann estimate = −4.9, 95% CI: −34.9 to 4.0; *r* = 0.41). On Day 4, the maximum observed in non-pregnant bitches was 14.0 pg/mL, compared with 29.8 pg/mL in pregnant bitches (*p* = 0.143; Hodges–Lehmann estimate = −11.7, 95% CI: −20.8 to 4.1; *r* = 0.53). Overall, the estimated marginal median over the entire observation period tended to be higher in the pregnant group than in the non-pregnant group (*p* = 0.068; Hodges–Lehmann estimate = −8.5, 95% CI: −15.9 to −3.6; *r* = 0.55).

**Table 1:** Serum kisspeptin concentrations (pg/mL) in pregnant and non-pregnant bitches at 15, 30, 45, and 60 days post-ovulation, expressed as median, minimum, and maximum.

Day post-ovulation	Group						*p*-value
	Non-pregnant			Pregnant			
	Median	Minimum	Maximum	Median	Minimum	Maximum	
15	9.1	3.8	15.9	20.7	19.9	33.2	0.029
30	14.9	7.4	18.1	19.5	9.4	37.9	0.486
45	8.5	5.6	16.2	13.1	9.1	40.7	0.486
60	11.3	8.9	14.0	23.2	10.0	29.8	0.143
Marginal median (overall period)	11.3	3.8	18.1	19.9	9.1	40.7	0.068

Marginal medians for the overall observation period are also reported. *p*-values refer to comparisons between groups, determined using the Independent-Samples Median Test.

No significant changes over time were observed within either the pregnant group (Friedman test, *χ*^2^(3) = 0.200, *p* = 0.978; Kendall’s *W* = 0.022) or the non-pregnant group (Friedman test, *χ*^2^(3) = 1.800, *p* = 0.615; Kendall’s *W* = 0.20).

## 4 Discussion

The kisspeptinergic system plays a key role in regulating reproductive function in mammals (46). To the best of our knowledge, this is the first study to evaluate serum kisspeptin concentrations in pregnant bitches compared with non-pregnant bitches in diestrus.

Our preliminary results showed a general tendency towards higher serum kisspeptin concentrations in pregnant dogs throughout the study period. While no significant temporal changes were detected within groups, a statistically significant difference was observed between pregnant and non-pregnant animals at 15 days post-ovulation. The substantial variability observed between individuals in both groups may reflect physiological factors unique to the canine species that influence kisspeptin secretion or metabolism.

The early increase in circulating kisspeptin concentrations observed in pregnant bitches may be biologically relevant as it coincides with the maternal recognition of pregnancy phase. Kisspeptins have been shown to regulate several essential mechanisms for establishing a pregnancy. In humans, placental kisspeptin production mainly occurs in syncytiotrophoblasts, while KISS1R is expressed in both cytotrophoblasts and syncytiotrophoblasts. This suggests that kisspeptins have autocrine and paracrine regulatory functions (31–35). Experimental evidence shows that kisspeptins promote embryo implantation by encouraging trophoblast adhesion to the endometrium and stimulating stromal decidualisation by increasing the production of leukaemia inhibitory factor (LIF) (31).

Additionally, kisspeptins modulate trophoblast behaviour, limiting excessive cellular migration and invasion, both of which are essential for proper placental development (31). They also play a role in regulating placental angiogenesis and spiral artery remodelling within the uterine microenvironment to ensure adequate maternal-fetal circulation (35–37). Furthermore, kisspeptins contribute to maternal immune tolerance by promoting the differentiation of naïve T cells into regulatory T cells (38). Studies have shown that KP-54 promotes the differentiation of regulatory T cells and inhibits the induction of T-helper 17 lymphocytes (47–49). Furthermore, exposure to kisspeptins reduces the cytotoxic activity of natural killer cells towards fetal cells, while decreasing neutrophil activity and increasing monocyte functional activity, thereby contributing to immune adaptation during pregnancy (49).

In addition to their physiological functions, kisspeptins have been suggested as potential biomarkers for various pregnancy-related complications, such as miscarriage, ectopic pregnancy, preterm birth, foetal growth restriction, hypertensive disorders of pregnancy, pre-eclampsia, gestational diabetes mellitus and gestational trophoblastic disease (50). These observations support the hypothesis that circulating kisspeptin concentrations may reflect placental functional activity.

Comparative studies across species further support the biological significance of kisspeptin during pregnancy. In humans, circulating kisspeptin concentrations increase dramatically throughout gestation. They reach levels approximately 900-fold higher than baseline during the first trimester and up to 7,000-fold higher during the third trimester. This is followed by a rapid return to normal levels after delivery (39). Similarly, studies in cattle have demonstrated rising plasma KP-10 levels during pregnancy (27). However, some studies have reported variable temporal profiles, including increases in early gestation, decreases in mid-gestation and subsequent increases in late pregnancy. This suggests that there are species-specific regulatory mechanisms for the secretion of placental kisspeptin (40).

Differences between our findings and those reported in other species may reflect variations in gestational length and sampling protocols. While kisspeptin concentrations in humans and cattle have been evaluated across broad gestational stages, blood samples in the present study were collected at specific time points (15, 30, 45, and 60 days post-ovulation). Furthermore, the canine reproductive cycle is characterised by a prolonged luteal phase and physiological diestrus occurs regardless of pregnancy status. This peculiar endocrine environment may account for the similarities observed between pregnant and non-pregnant animals and may partially explain the limited statistical differences seen at later time points.

From a clinical perspective, the early increase in kisspeptin concentrations observed in pregnant dogs suggests that kisspeptin may be an endocrine signal associated with the early stages of pregnancy in female dogs. Currently, ultrasonography is the earliest reliable diagnostic tool, enabling pregnancy to be detected from around 19 days post-ovulation. Therefore, if confirmed in larger prospective studies, measuring circulating kisspeptin levels could help to improve the monitoring of early pregnancy in reproductive management.

Ongoing studies involving larger populations of animals are currently underway to investigate the gestational pattern of serum kisspeptin concentrations further and to evaluate the clinical application of kisspeptin as an early pregnancy biomarker. Given the well-established role of kisspeptins as biomarkers of pregnancy-related complications in humans (50), it is reasonable to hypothesise that circulating kisspeptin concentrations could also serve as potential indicators of gestational pathologies in dogs. However, further research is needed to confirm this.

The present study has several limitations that should be acknowledged. These include the extremely limited sample size, the fact that only one breed was included, and the relatively wide sampling intervals adopted in the study design. Furthermore, the absence of diagnostic performance analyses, such as sensitivity-specificity assessments or receiver operating characteristic (ROC) curve modelling, means that the present findings cannot be applied clinically. It should also be emphasised that only circulating kisspeptin concentrations were evaluated, while placental expression and underlying mechanistic pathways were not investigated. Therefore, the proposed biological interpretations remain speculative. Future studies integrating molecular placental investigations with larger, more heterogeneous populations are essential for better clarifying the physiological significance and potential clinical relevance of kisspeptins during canine pregnancy.

## Data Availability

The original contributions presented in the study are included in the article/supplementary material, further inquiries can be directed to the corresponding author.
